# Publications Received

**Published:** 1871-11

**Authors:** 


### PUBLICATIONS RECEIVED.

The Functions and Disorders of the Reproductive Organs in Childhood, Youth, Adult Age, and Advanced Life, Considered in their Physiological, Social and Moral Relations. By William Acton, M.R.C.S., Late Surgeon to the Islington Dispensary, and formerly Externe to the Venereal Hospitals, Paris; Fellow of the Royal Medical and Chirurgical Society, etc. Third American, from the Fifth London Edition. Philadelphia: Lindsay & Blakiston. 1871.

Mr. Acton has treated the various subjects embraced in this work in an elaborate and philosophical spirit, raising them from the platform of passion and sensuality to the higher basis of a truly scientific investigation. He treats specially of the functions and disorders as distinguished from the anatomy and pathology of the reproductive organs. He has laid under contribution the domains of natural history and comparative anatomy, with the illustrative treasures of the College of Surgeons' Museum, the Veterinary College, and the Zoological Gardens, as well as availed himself of the experience of the practical breeders of stock.

Taking each of the periods of life separately, he has first discussed the normal functions or conditions of the reproductive organs incidental to it. Having thrown the light of the most recent physiological investigations upon these, he has discussed the disorders to which each period is most subject, and has embraced the whole of the diseases to which the generative functions are regarded as liable, and in addition has indicated the treatment supposed to be appropriate to each. We have no hesitation in asserting that the work is a very able one, and will do much to wrest the disorders of the reproductive organs from the grasp of quackery, and arouse a greater zeal in the effort to relieve this class of troubles, upon the part of the scientific and conscientious practitioner.

Practical Midwifery and Obstetrics, Including Anæsthetics. By John Tanner, M.D., M.A., LL.D., London; Member of the Royal College of Physicians; Obstetric Physician to the Farrington Lying-in Charity, etc, Philadelphia: J. B. Lippincott & Co. London; J. & A. Churchill. 1871.

The object of the author is to afford a concise and clear description of the Science of Obstetrics, and to furnish a complete hand-book to this science; and he asserts that by its aid the student will be enabled to go to the bedside of the lying-in for the *first time* with perfect confidence in himself, and without being considered an amateur either by nurse or patient; and the qualified practitioner, by reference to its pages and diagrams, can in a minute or two refresh his memory, and undertake the most difficult obstetric case, with its attendant consequences and complications.

The work professes to contain not only everything of importance that is to be found in other books upon the subject, but is supplemented by facts and incidents experienced by the author during an extensive public and private practice.

The author does not touch upon the anatomy and physiology of the organs of generation, regarding it as of more value to devote the work to the consideration of Midwifery alone, in a practical and thorough manner.

Some of the illustrations are modifications of those found in other works, but many of them are original, exhibiting actual occurrences in the experience of the author.

The work doubtless will be highly valued by those who desire books of the manual class.

THE AMERICAN JOURNAL OF OBSTETRICS AND DISEASES OF WOMEN AND CHILDREN. Edited by B. F. DAWSON, M.D., Attending Physician to the Out-Door Department of the New York State Women's Hospital; to the New York Free Dispensary for Sick Children; and Assistant to the Clinique for Diseases of Children in the College of Physicians and Surgeons, New York. Published by Wm. Baldwin & Co., 21 Park Row, New York, opposite the Astor House, to whom all Communications for the Journal or Editor are to be directed.

We have received two bound volumes of this work, containing a large mass of valuable material, besides the quarterly number for August, 1871, in which we find a large number of original communications from able writers in Europe and this country, among which, as worthy of special notice, we find:

"Sudden Death soon after Parturition." By Thomas Moore Madden, M.D., M.R.I.A, Dublin, Ireland; Licentiate of the College of Physicians, Ireland; Member of the Royal College of Surgeons of England, etc.

"Retrospect of Uterine Pathology and Therapeutics in the

United States; Specially in Regard to Intra-Uterine Medication in Chronic Internal Metritis." By Henry Miller, M.D., Louisville, Kentucky, Emeritus Professor of Obstetrics and Diseases of Women and Children in the Louisville Medical College, and President of the Faculty, etc.

"The Mechanical Treatment of Displacements of the Unimpregnated Uterus." By George Pepper, M.D., Philadelphia, Pennsylvania.

"Description of a new Speculum Vaginæ, with Cases Illustrating its Practical Value in Uterine Surgery." By J. Byrne, M.D., Brooklyn, Surgeon to St. Mary's Hospital for Diseases of Women.

"Ovarian Dropsy of Fifty Years' Standing—the Patient being Three Times Pregnant during its Existence, and giving Birth to Living Children on each Occasion." By Robert P. Harris, M.D., Philadelphia, Pennsylvania.

"A New Ovariotomy Clamp." By B. F. Dawson, M.D., Editor, New York.

"The Use of Electricity in the Treatment of the Diseases of Children." By Dr. Ullers-Berger, Paris, France. A Prize Essay, to which was awarded the prize of one hundred dollars in gold offered by the Editors ot the Journal.

In addition, it also contains a department of "Clinical Notes on Diseases of Children," "Transactions of the New York Obstetrical Society," "Transactions of the Philadelphia Obstetrical Society," and "A Quarterly Report on Obstetrics and Diseases of Women and Children."

Terms, $5 a year; single numbers, $1.50. A work which should be in the hands of every practitioner.

We have also received the following:

*Medical and Surgical Electricity.* By Geo. M. Beard, A.M., M.D., Fellow of the New York Academy of Medicine, etc., and A. D. Rockwell, A.M., M.D., Fellow of the New York Academy of Medicine, etc. With One Hundred and Two Illustrations. Published by Wm. Wood & Co., New York.

*A System of Surgery.* By T. Holmes, M.A., Cantab. Surgeon and Lecturer on Surgery at St. George's Hospital. Published by Wm. Wood & Co., New York.

*Selected Obstetrical and Gynæcological Works of Sir James Y. Simpson, Bart., M.D., D.C.L., Late Professor of Midwifery in the University of Edinburgh.* Edited by J. Watt Black, M.A., M.D., Member of the Royal College of Physicians, London, etc. Published by D. Appleton & Co., New York.

*A Treatise on Diseases of the Nervous System.* By William A. Hammond, M.D.. Professor of the Diseases of the Mind and Nervous System, and of Clinical Medicine, in the Bellevue Hospital Medical College, etc. With Forty-Five Illustrations. Published by D. Appleton & Co., New York.

*General Surgical Pathology and Therapeutics.* By Dr. Theodore Billroth, Professor of Surgery in Vienna. Translated from the German by Charles E. Hackley, A.M., M.D., Surgeon to the New York Eye and Ear Infirmary, etc. Published by D. Appleton & Co., New York.

*Practical Therapeutics.* By Edward John Waring, M.D., F.L.S., Member of the Royal College of Physicians, London, etc. Published by Lindsay & Blakiston, Philadelphia.

*The Druggist's General Receipt Book.* By Henry Beasley, Author of the "Book of Prescriptions," etc. Published by Lindsay & Blakiston, Philadelphia.

*Emergencies, and How to Treat Them.* By Joseph W. Howe, M.D., Visiting Surgeon to Charity Hospital, Lecturer on Surgery in the Medical Department of the University of New York. Published by D. Appleton & Co., New York.

*Skin Diseases: Their Description, Pathology, Diagnosis and Treatment.* By Tilbury Fox, M.D., London, M.R.C.P., Fellow of the University College, Physician to the Skin Department of University College Hospital. Edited by M. H. Henry, M.D., Surgeon to the New York Dispensary—Department of Venereal and Skin Diseases. Published by Wm. Wood & Co., New York.

*Galvano-Therapeutics: The Physiological and Therapeutical Action of the Galvanic Current upon the Acoustic, Optic, Sympathetic and Pneumogastric Nerves.* By William B. Neftel, M.D. Published by D. Appleton & Co., New York.

*Prolapsus Uteri—Its Chief Causes and Treatment.* By Thomas Addis Emmett, Surgeon-in-Chief of the Women's Hospital of the State of New York. Published by Bradstreet Press, New York.

*Amputation of Redundant Scrotum in the Treatment of Varicocele.* Illustrated with a new Instrument. By M. H. Henry, M.D., Surgeon to the New York Dispensary—Department of Veneral and Skin Diseases, etc.

*Transactions of the Americal Otological Society.* Third Annual Meeting, Newport, R. I., July 20, 1870. Published by D. Appleton & Co., New York.

*Physical Effects of Compressed Air, and of the Causes of Pathological Symptoms Produced on Man by Increased Atmospheric Pressure Employed for the Sinking of Piers in the Construction of the Illinois and St. Louis Bridge, over the Mississippi River at St. Louis, Missouri.* By A. Jaminet, M.D. Published by R. & T. A. Ennis, St. Louis, Missouri.

*Proceedings of the State Medical Association of Arkansas, at Little Rock, November,* 1870.

*An Address Decivered Before the Alumni Association, at the Jefferson Medical College, at its First Anniversary.* By S. D. Gross, M.D., LL.D., Professor in the College and President of the Association.

*Description of the Orthopœdic Apparatus Employed in the Treatment of Deformities and Deficiencies of the Human Body, with Directions for Taking Measurements for Their Application.* By D. W. Kolbe, Manufacturer of Surgical Instruments, and Mechanist to the Philadelphia Orthopœdic Hospital.

*Fortieth Annual Announcement of the Medical College of Georgia, Augusta—Session* 1871–72.

*Thirty-Fifth Annual Announcement of the Medical Department of the University of Louisville—Session of* 1871–72.

*Twenty-Ninth Annual Announcement of Rush Medical College, Chicago, Illinois—Session* 1871–72.

*The Twenty-Second Annual Announcement of Lectures, University of Nashville, for the Session of* 1871–72. Published by Paul & Tavel, Nashville, Tenn.

*Second Annual Report of the Maryland Eye and Ear Infirmary, Baltimore, Maryland.* Published by James Young.

*A Case of Skin-Grafting.* By John W. Trader, M.D., Sedalia, Missouri. Published by Levison & Blythe, St. Louis, Missouri.

